# Potential of Lactic Acid Bacteria Isolated From Different Forages as Silage Inoculants for Improving Fermentation Quality and Aerobic Stability

**DOI:** 10.3389/fmicb.2020.586716

**Published:** 2020-12-08

**Authors:** Melisa Puntillo, Mónica Gaggiotti, Juan Martín Oteiza, Ana Binetti, Ariel Massera, Gabriel Vinderola

**Affiliations:** ^1^Instituto de Lactología Industrial (CONICET-UNL), Facultad de Ingeniería Química, Universidad Nacional del Litoral, Santa Fe, Argentina; ^2^Laboratorio de Calidad de Leche y Agroindustria, INTA EEA Rafaela, Santa Fe, Argentina; ^3^Centro de Investigación y Asistencia Técnica a la Industria, Río Negro, Argentina

**Keywords:** lactic acid bacteria, silage, inoculant, stability, fermentation

## Abstract

We aimed at isolating lactic acid bacteria (LAB) from different plant materials to study their crossed-fermentation capacity in silos and to find strains able to confer enhanced aerobic stability to silage. A total of 129 LAB isolates were obtained from lucerne (alfalfa), maize, sorghum, ryegrass, rice, barley, canola, *Gatton panic*, *Melilotus albus*, soy, white clover, wheat, sunflower, oat, and moha. Four *Lactiplantibacillus plantarum* subsp. *plantarum* strains (isolated from oat, lucerne, sorghum, or maize) were selected for their growth capacity. Identity (16S sequencing) and diversity (RAPD-PCR) were confirmed. Fermentative capacity (inoculated at 10^4^, 10^5^, 10^6^, 10^7^ CFU/g) was studied in maize silage and their cross-fermentation capacity was assessed in oat, lucerne, sorghum, and maize. Heterofermentative strains with the highest acetic acid production capacity conferred higher aerobic stability to maize silages. Regardless the source of isolation, *L. plantarum* strains, inoculated at a rate of 10^6^ CFU/g, were effective to produce silage from different plant materials. From more than 100 isolates obtained, the application of a succession of experiments allowed us to narrow down the number of potential candidates of silage inoculants to two strains. Based on the studies made, *L. plantarum* LpM15 and *Limosilactobacillus fermentum* LfM1 showed potential to be used as inoculants, however further studies are needed to determine their performance when inoculated together. The former because it positively influenced different quality parameters in oat, lucerne, sorghum, and maize silage, and the latter because of its capacity to confer enhanced aerobic stability to maize silage. The rest of the strains constitute a valuable collection of autochthonous strains that will be further studied in the future for new applications in animal or human foods.

## Introduction

Preserved forages, such as silages, are used as a major proportion of the diet fed to dairy cows and other cattle in many milk and meat producing countries. *Lactiplantibacillus plantarum* subsp. *plantarum* (formerly *Lactobacillus plantarum*, Zheng et al., 2020), *Pediococcus pentosaceus* and *Enterococcus faecium* are probably the homofermentative lactic acid bacteria (LAB) species most intensively used as silage inoculants (Ogunade et al., 2019). When silos are opened and the ensiled material is exposed to the air, a deterioration process may begin, mainly by lactate assimilating yeasts, which leads to an increase in the silage temperature followed by an increase in the pH values, allowing the growth of other aerobic microorganisms and further deterioration (Kung et al., 2018). In order to overcome this problem, heterofermentative LAB, mainly *Lentilactobacillus buchneri* subsp. *buchneri*, have been used for more than two decades so far for silage preservation (Filya, 2003; Schmidt et al., 2009). In common daily use of silage inoculants, some issues related to the concentration of viable LAB to be applied, the efficacy of strains isolated from plant materials different to the one to be fermented and the possibility of using heterofermentative species other than *L. buchneri* for enhanced aerobic stability, are of interest. Isolation of novel LAB strains for application in silage has been a common practice over the years but it is still an activity with current importance around the globe (dos Santos Leandro et al., 2020; Paradhipta et al., 2020; Tanizawa et al., 2020), due to the interest in collecting diverse strains for future applications not only as silage inoculants but also in other plant-based food for animals and humans use (Wuyts et al., 2020). In Argentina, the ensiling market is highly dominated by products manufactured abroad with foreign strains, being the European countries the ones leading the market with a share of 44% of the global silage additives market (Fabiszewska et al., 2019). The availability of strains locally sourced will foster the development of products that may fuel our compromised economy. In addition, the key inoculant companies in the market are expanding their business units in various geographical areas, focusing on agreements and partnerships with local players and distributors, including researchers, introducing new effective products through investments in R&D (Fabiszewska et al., 2019). Then, the importance of local research and evaluation of new strains adapted to the fermentative process of local forages was recently highlighted (Amaral et al., 2020).

The aim of this work was to isolate LAB strains from different plant materials to study their crossed-fermentation capacity in silos and to find novel candidates to promote aerobic stability to be exploited in the southern cone. To the best of our knowledge, this is the study where the most diverse variety of forages was used within the same work (15 different plant materials), to isolate new LAB strains for preliminary characterization and future exploitation.

## Materials and Methods

### Sample Collection

The following plant materials were sampled: lucerne (alfalfa: *Medicago sativa*), maize (*Zea mays*), sorghum (*Sorghum* spp.), ryegrass (*Lolium perenne*), rice (*Oryza sativa)*, barley (*Hordeum vulgare*), canola, *Gatton panic*, *Melilotus albus*, soy (*Glycine max*), white clover (*Trifolium repens*), wheat (*Triticum* spp.), sunflower (*Helianthus annuus*), oat (*Avena sativa*), and moha (*Setaria italica*). Samples were obtained in duplicates from local farmers of Santa Fe Province (cities of Esperanza, Rafaela, Cavour, Grutly, Frank, Pozo Borrado, and Candioti) and Buenos Aires Province (cities of Pergamino and Tandil) during 2016. Samples (1 Kg) were harvested at an adequate physiological state for silage, indicated by the local farmers, and immediately transported to the laboratory, where they were chopped to approximately 1 cm using a laboratory forage chopper (BIMG-METVISA, Brazil), except for maize and sorghum, which were chopped at the farmer’s place, by a farming chopper. After chopping, the material was ensiled using a small-scale system of silage fermentation, as follows: approximately 500 g portions of each material were packed into polyethylene bags (Cryovac: BB4LA), sealed with a vacuum sealer (Turbovac, Bosch) and incubated at 34°C (MiLab, SPX-250 B III, China). Samples were allowed to ferment until a pH lower than 4.5 was achieved (Orion 3 Star, Thermo Fisher Scientific, Beverly, MA, United States), measuring pH in several replicates of the ensiled material, for successive measurements until pH 4.5 was observed.

### Isolation of LAB

**FIGURE 1:**
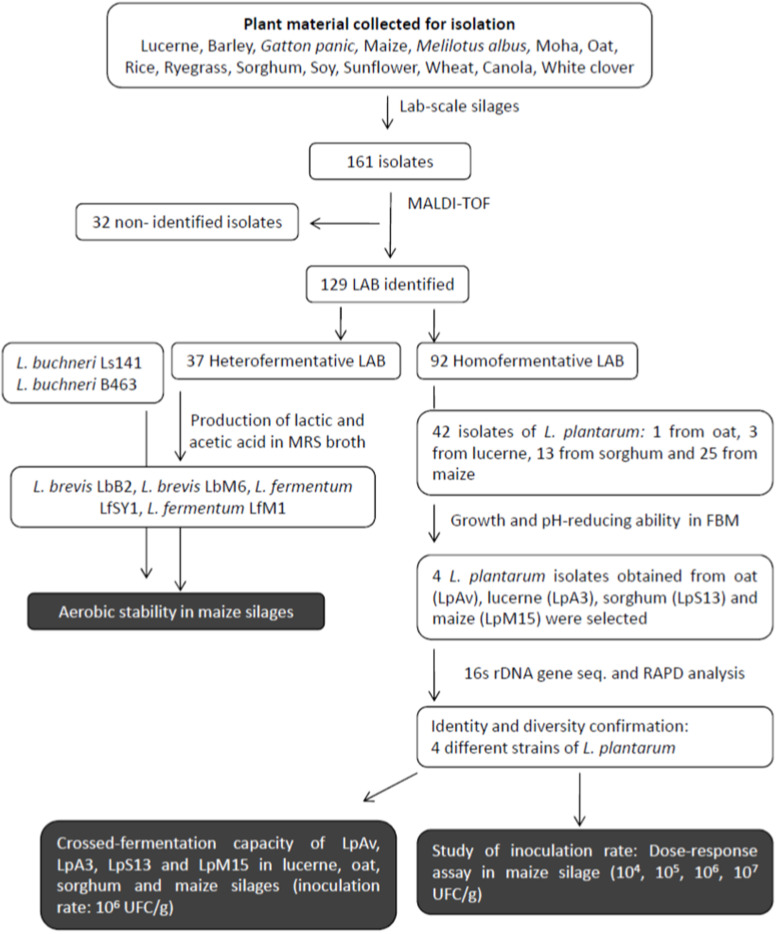
Flow chart of the isolation, selection, and characterization process.

Figure 1 shows the flow chart of the isolation, selection, and characterization processes used in this study. A sample (10 g) of the fermented plant material was aseptically homogenized with 90 ml of sterile 0.85% NaCl solution in a sterile plastic bag (Nasco WHIRL-PACK, United States) using a stomacher (3 min, high speed, three cycles). Supernatants were serially diluted with peptone water (0.1% w/v, Britania, Buenos Aires, Argentina) and surface-plated on MRS agar (Biokar, Beauvais, France). Plates were incubated (aerobiosis, 34°C, 48 h). Colonies presenting typical LAB morphology (immersion microscopy, 1000×) were picked-up and streaked on MRS agar. Gram-staining, mobility, catalase activity, spore-forming capacity, and gas production from glucose in MRS broth (Dürham tubes) were assessed using standard procedures. Presumptive homo and heterofermentative LAB isolates were frozen-stored (at −20°C and −70°C) in MRS-20% (v/v) glycerol broth. The term presumptive was used until proper identification was carried out.

### Identification of Isolates

Preliminary identification was carried out according to the protein and peptide profile by means of Matrix-Assisted-Laser-Desorption-Ionization-Time-of-Flight Mass Spectrometry (MALDI-TOF MS), using an Axima Performance mass spectrometer (Shimadzu Scientific Instruments, United States) in conjunction with the commercial Spectral Archive And Microbial Identification System (SARAMIS) database. Analyses were performed according to the manufacturer’s instructions using the direct smear technique, by the Mass Spectroscopy Laboratory from the Biological Sciences and Biochemistry Faculty of the National University of Litoral (Santa Fe, Argentina).

### Growth Kinetics and Selection of *L. plantarum* Strains

#### Growth Kinetics in Forage-Based Medium

Forage-based media (FBM) were prepared with lucerne, oat, sorghum, and maize (ABM, OBM, SBM, MBM, respectively). Fresh (non-fermented) samples of chopped lucerne, oat, sorghum, or maize were mixed (1:10) with distilled water and homogenized (stomacher, 3 min, high speed, three cycles). The suspension was filtered (filter papers Quanty JP41 Faixa Preta, Londrina, PR, Brazil) and centrifuged (5000 × *g*, 10 min, 8°C). The pH of the supernatant was adjusted to 6.5 with 1 M NaOH, aliquoted and autoclaved (121°C, 15 min). Each FBM was inoculated (1% v/v) with an overnight culture (MRS broth, adjusted to 1 × 10^8^ CFU/ml) of each *L. plantarum* strain isolated from oat, lucerne, sorghum, and maize. All isolates were tested at this stage. Cultures were previously washed twice with PBS (phosphate buffered saline solution, pH 7.4) in order not to carry over nutrients from MRS broth. Inoculated FBM (ca. 10^6^ CFU/ml) were distributed (300 μl/well) in 96-well microplates (Thermo Scientific Multiskan FC Microplate Photometer) and incubated at 34°C in aerobiosis. Optical density (OD_560 *nm*_) was measured every 30 min during 18 h. In parallel, a sample of inoculated FBM (ca. 10^6^ CFU/ml) was incubated in 5 ml test tubes (aerobiosis, 34°C for 24 and 48 h). OD_560 *nm*_, pH and cell counts (MRS agar) were performed. Each strain was assayed in independent triplicates. Results were expressed as (Δlog_10_ CFU/ml), where Δ is the difference between cell counts after 24 or 48 h with respect to the initial count.

#### Identity Confirmation

Total DNA of selected *L. plantarum* strains was extracted from overnight cultures (18 h) by using GenElute Bacterial Genomic DNA kit (Sigma, St. Louis, MO, United States) according to the manufacturer’s instructions. Purified DNA samples were stored at −20°C until use. The identity of isolates was analyzed by amplifying, sequencing and comparing a 1500 bp fragment within their 16S rRNA gene (pA: AGA GTT TGA TCC TGG CTC AG, pH: AAG GAG GTG ATC CAG CCG CA) (Edwards et al., 1989). All PCR reactions were performed using 2 μl of diluted (1:50) DNA as template, 2.5 U Taq DNA polymerase (GE Helathcare, Little Chalfont, United Kingdom), 200 μM dNTPs (GE Healthcare) and 100 nM each primer (Sigma-Genosys, The Woodlands, TX, United States) in a final volume of 50 μl. Amplifications were performed in a GeneAmp PCR System (Applied Biosystem, Foster City, CA, United States) under the following conditions: 3 min at 94°C, 36 cycles of denaturation at 94°C for 1 min, 2 min annealing at 51°C and 2 min extension at 72°C, and a final step of 7 min at 72°C. The PCR products were separated on 0.8% (w/v) agarose gels in TBE buffer, stained with GelRed (Biotium, Hayward, CA, United States) and visualized under UV light. Amplicons were purified with MicroSpin Columns (GE Healthcare) and their nucleotide sequences were determined by primer extension at the DNA Sequencing Service of Macrogen (Seoul, South Korea). The identity of isolates was checked by nucleotide-nucleotide BLAST of the NCBI database^1^.

#### RAPD Analysis

The genotypic diversity of selected *L. plantarum* isolated was analyzed by RAPD-PCR, using two arbitrary primers, B10 (5′- CTGCTGGGAC -3′) and M13 (5′- GAGGGTGGCGGTTCT -3′), and amplification conditions were primer-dependent (Giraffa et al., 2004; Binetti et al., 2007).

### Lab-Scale Silage Preparation

The following general procedure for micro-silos manufacture was used along this work to study the ensiling capacity of selected LAB strains. Overnight cultures of the strains used in the experiments described below were obtained in MRS broth, centrifuged (5000 × *g*, 15 min, 8°C), washed twice with PBS (pH 7.2) and resuspended in NaCl 0.85% (w/v). Silages were made using the corresponding fresh-cut forage, employing a small-scale system for silage preparation (Burns et al., 2018). Growth stage at harvesting, for each forage, was the proper one for ensiling according to the farmer that provided the material. Forage was chopped (length of 17 mm) by a precision chop forage harvester (Claas Jaguar, Claas Group, Harsewinkel, Germany). Lucerne was left to wilt for 4 h before chopping. Chopped material was transported in plastic bags within 1 h of chopping to the laboratory and processed immediately. Sixty kilogram of chopped forage were sprayed at a rate of 20 ml/kg of fresh forage, with the suspension of the strain at different concentrations (depending on the experimental design: 2.6.1, 2.6.2, or 2.7.2). Control (non-inoculated) samples were sprayed with the same amount of NaCl 0.85% w/v, which was used for preparing the strains suspensions. All treatments were applied at the same time, thanks to the aid of the laboratory personnel. Inoculated plant-material was distributed into 1 kg portions in polyethylene bags in triplicates for each sampling point (Cryovac: BB4LA) and vacuum-sealed (Turbovac, Bosch) to become a micro-silo. Micro-silos were kept at 25°C for different periods, depending on the specific assay (indicated below).

### *L. plantarum* Strains as Inoculants in Lab-Scale Silages

#### Dose-Response in Maize Silage

Four *L. plantarum* strains (one isolated from each substrate) were selected based on their growth kinetics on FBM. *L. plantarum* LpAv, LpA03, LpS13, and LpM15 were isolated from oat, lucerne, sorghum, and maize, respectively. Fresh maize silages (in triplicate, for each sampling day) were prepared as described before. The strains were inoculated at a rate of 10^4^, 10^5^, 10^6^, and 10^7^ CFU/g of fresh plant material. Control samples were sprayed with NaCl 0.85% w/v. Total LAB count and pH were determined in triplicates after 0, 24, 48, 72 h, and 30 days of fermentation.

#### Crossed-Fermentation Capacity in Lucerne, Oat, Sorghum, and Maize Silage

Lucerne, oat, sorghum, and maize silages were prepared as described above using four *L. plantarum* strains (LpAv, LpA03, LpS13, and LpM15). Each strain was used on each plant material (crossed-fermentation). Strains were inoculated at a rate of 1 × 10^6^ CFU/g of fresh chopped material. Control was sprayed with the same volume of NaCl 0.85% w/v. Microbiological analyses and pH determinations were carried out at different days of storage, depending on the forage: 0, 2, and 30 days (oat); 0, 3, and 30 days (lucerne); 0, 1, 3, and 30 days (sorghum and maize), as suggested by the farmers that provided each material. Total LAB were enumerated in triplicates MRS agar (34°C, 48 h, aerobiosis) and yeasts and molds in chloramphenicol glucose agar (Biokar, Beauvais, France) (25°C, 7 days, aerobiosis).

The following parameters were assessed, in triplicates, at the beginning and after 30 days of fermentation: Dry Matter (% DM; PROMEFA-v2 AOAC, 1990 N° 130.15 and N° 167.03), Crude Protein (% CP; AOAC, 1998 N° 976.05), Ash (% Ash, AOAC 1990 N1C 942.05), Acid Detergent Fiber (% ADF; ANKOM Method validated with ISO13906:2008), Neutral Detergent Fiber (% NDF; ANKOM Method validated with ISO16472:2006), Acid Detergent Lignin (% LDA; PROMEFA-v2, ANKOM), Ether Extract (% EE; AOAC 1999 N° 920.39). Ammonia Nitrogen/Total Nitrogen (N NH3/N T; Blain and Urtunette, 1954) was quantified after different days of fermentation: 2 and 30 days (oat); 3 and 30 days (lucerne); 1 and 30 days (sorghum and maize). Analyses were performed by the Laboratorio de Forrajes, Instituto Nacional de Tecnología Agropecuaria (INTA, Rafaela, Santa Fe, Argentina). Analyses were carried out in triplicate.

### Selection of Heterofermentative LAB for Enhanced Aerobic Stability

#### Lactic and Acetic Acid Quantification in Heterofermentative Strains

The 37 heterofermentative LAB isolated in this study were inoculated (1% v/v, in triplicates) on 10 ml of MRS broth supplemented with 0.1% (w/v) cysteine. *L. buchneri* Ls141 and 463 were used as external reference strains. *L. buchneri* Ls141 had been isolated from maize silage in a previous work (Burns et al., 2018) and it is currently used in a commercial inoculant in Argentina. *L. buchneri* 463 was isolated from a spoiled commercial tomato sauce (the strain was kindly provided by Dr. Juan Martín Oteiza). After 72 h of anaerobic incubation at 34°C, cultures were centrifuged (5000 × *g*, 10 min, 8°C) and supernatans were filtered (0.45 μm cellulose nitrate filter, Sartorius, Germany). Quantification of lactic and acetic acids was performed by HPLC. Chromatographic separation was carried out isocratically at 65°C with a mobile phase of 10 mM H_2_SO_4_ at a flow rate of 0.6 ml/min on an Aminex HPX-87H column (300 × 7.8 mm) equipped with a cation H+ microguard cartridge (Bio-Rad Laboratories, United States). The supernatant of cultures after centrifugation was diluted 1:3 with 10 mM H_2_SO_4_, filtered through 0.45 μm membranes (Millex, Millipore, Brazil) and injected into the chromatograph, using a loop of 60 μl. HPLC equipment consisted of a quaternary pump, an on-line degasser, a column oven, a UV-visible detector (all Series 200) and a refractive index detector thermostatised at 35°C (Series Flexar) (Perkin Elmer, United States). The UV detector was set at 210 nm for the detection of organic acids. Data were collected and processed on a computer with the software Chromera (Perkin Elmer).

#### Aerobic Stability in Maize Silage

In order to study the capacity of selected heterofermentative strains to control yeasts and molds, fresh-cut maize silages were prepared as described before. *Limosilactobacillus fermentum* LfSY and LfM1, *Levilactobacillus brevis* LbB2 and LbM6 and *L. buchneri* Ls141 and 463 were inoculated at a rate of 1 × 10^6^ CFU/g of fresh chopped material. A control (non-inoculated) was included. The pH values and LAB counts were determined, in triplicates, at the beginning (*t* = 0) and after 90 days of fermentation. Dry Matter (DM) was determined as indicated above. After 90 days, silages were opened and aerobic stability was determined according to Burns et al. (2018) in a room were temperature was controlled at 21 ± 1°C, in duplicates. Aerobic stability was defined as the time necessary for the internal temperature of silage to increase 2°C above room temperature.

### Statistical Analyses

All measurements were performed in triplicate and the results were presented as mean ± standard deviation. Data were analyzed using the one-way ANOVA procedure of SPSS 15.0 software (SPSS Inc., Chicago, IL, United States). The differences between means were detected by Tukey or Dunnett test, depending whether comparison was among all groups (Tukey) or compared to the control group (Dunnett). Differences were considered statistically significantly different when *p* < 0.05.

## Results

### Isolation and MALDI-TOF Identification of LAB From Different Plant Material

Fifty-one samples from 15 forage crops were processed. Except from Canola, a total of 161 presumptive LAB isolates were obtained from the other 14 forage crops. Table 1 shows the identity (by MALDI-TOF) and origin of the 129 isolates identified as belonging to different species of the LAB group, the rest of the isolates were not identified by MALDI-TOF, and were no longer considered for this study. *L. pentosus/plantarum* was the homofermentative species for which the highest number of isolates were obtained. The most frequently isolated heterofermentative species was *L. brevis*, which was found in 6 out of 14 plant materials studied.

**TABLE 1:** Identity (MALDI-TOF MS) of LAB isolates derived from plant material.

Plant material^*a*^	Isolated species	Isolate identification code
Lucerne (5)	*L. pentosus/plantarum*	LpA1, LpA2, LpA3
	*Pediococcus acidilactici*	PaA1, PaA2, PaA3, PaA4
	*P. pentosaceus*	PpA1, PpA2
Barley (5)	*L. brevis*	LbB1, LbB2, LbB3
	*Lactococcus garvieae*	LgB1, LgB2
	*Enterococcus hirae*	EB1, EB2
	*Enterococcus avium/Enterococcus raffinosus*	EB3
	*E. faecium*	EB4
*Gatton panic* (2)	*Enterococcus durans*	EG1
Maize (11)	*L. pentosus/plantarum*	LpM0, LpM1, LpM2, LpM3, LpM4, LpM5, LpM6, LpM7, LpM8, LpM9, LpM10, LpM11, LpM12, LpM13, LpM14, LpM15, LpM16, LpM17, LpM18, LpM19, LpM20, LpM21, LpM22, LpM23, LpM24
	*L. paracasei*	LpaM1
	*L. brevis*	LbM1, LbM2, LbM3, LbM4, LbM5, LbM6, LbM7, LbM8, LbM9, LbM10, LbM11, LbM12, LbM13, LbM14, LbM15, LbM16, LbM17
	*L. fermentum*	LfM1, LfM2, LfM3, LfM4
	*L. rhamnosus*	LrM1
	*P. acidilactici*	PaM1
	*P. pentosaceus*	PpM1, PpM2, PpM3
	*Leuconostoc citreum*	LciM1
	*Weissella confusa*	WcM1
	*Lactobacillus* sp.	LspM
*Melilotus albus* (1)	*L. pentosus/plantarum*	LpML1
	*P. pentosaceus*	PpML1
Moha (1)	*L. fermentum*	LfMH
Oat (5)	*L. pentosus/plantarum*	LpAv
	*L. paracasei*	LcAv
	*L. brevis*	LbAv
	*P. acidilactici*	PaAv1, PaAv2, PaAv3
	*Leuconostoc pseudomesenteroides*	LeuAv
Rice (3)	*L. fermentum*	LfRi1
Ryegrass (1)	*L. pentosus/plantarum*	LpRY1, LpRY2, LpRY3, LpRY4, LpRY5
	*L. brevis*	LbRY1, LbRY2, LbRY3
	*W. confusa*	WcRY1
Sorghum (6)	*L. pentosus/plantarum*	LpS1, LpS2, LpS3, LpS4, LpS5, LpS6, LpS7, LpS8, LpS9, LpS10, LpS11, LpS12, LpS13
	*L. brevis*	LbS1
Soy (2)	*L. pentosus/plantarum*	LpSY1
	*L. brevis*	LbSY1
	*L. fermentum*	LfSY1, LfSY2, LfSY3, LfSY4
	*P. acidilactici*	PaSY1
Sunflower (3)	*L. pentosus/plantarum*	LpSF1
	*E. hirae*	EhSF1
Wheat (3)	*L. pentosus/plantarum*	LpW1, LpW2
	*L. pseudomesenteroides*	LeuW1, LeuW2
	*Lactococcu lactis*	LlW1
White clover (3)	*L. pentosus/plantarum*	LpWC1, LpWC2
	*E. faecium*	EfWC1, EfWC2
	*Enterococcus* sp.	EspWC1, EspWC2, EspWC3

*^*a*^The number between brackets indicates the number of samples analyzed.*

### Growth Capacity of *L. plantarum* in Forage-Based Medium: Selection and Identity Confirmation of Strains for Further Studies

**FIGURE 2:**
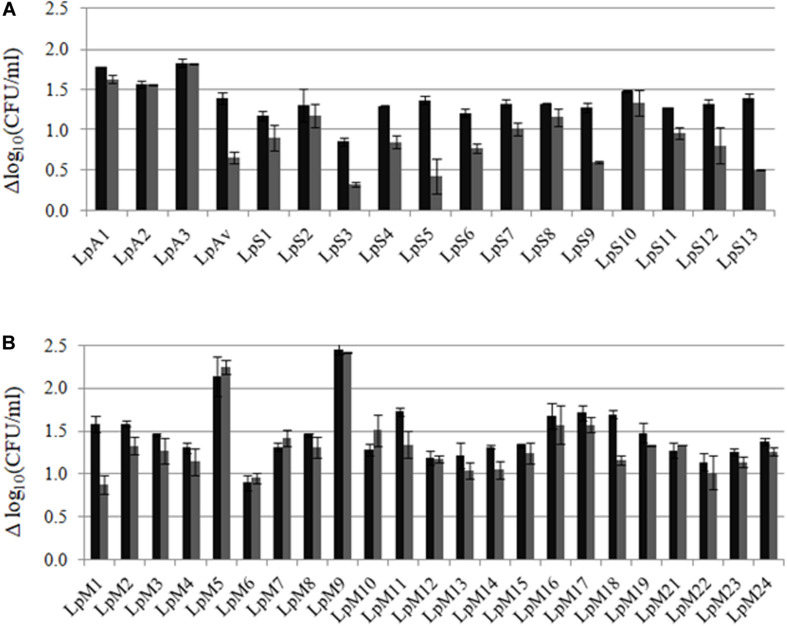
Growth (Δlog_10_ CFU/ml ± SD) of *L. plantarum*, isolated from lucerne, oat, sorghum and maize, after 24 (■) and 48 (■) h of culture in **(A)** lucerne-based medium (LpA1-LpA3), oat-based medium (LpAv), sorghum-based medium (LpS1-LpS13) and **(B)** maize-based medium (LpM21-LpM24). Values are means (±SD) of three repetitions.

The growth ability of *L. plantarum* strains isolated from lucerne, oat, sorghum or maize was assessed in the same FBM from which isolates were obtained: ABM, OBM, SBM, and MBM. Growth kinetics showed that the different isolates obtained from the same plant material displayed different growth capacity in laboratory-prepared media (growth kinetics, pH, and O.D. not shown). Figure 2 shows the differences in cell counts after 24 and 48 h of incubation in each FBM, compared to counts at time zero (Δlog_10_ CFU/ml). Most isolates grew from 1 to 1.5 log orders, while some isolates obtained from maize were able to reach the expected 2 log orders of growth in MBM (as 1% v/v inoculum was used). After 24 h of culture, loss of cell viability was observed in most cases.

Changes in the pH of the inoculated media showed the ability of all *L. plantarum* isolates to ferment the corresponding FBM. In OBM, LpAv dropped the pH from 6.55 to 5.80 (24 h) and 4.89 (48 h). In ABM, LpA1, LpA2, and LpA3 decreased the pH from 6.26 to 5.80–5.95 after 24 h of fermentation, and there was no further pH reduction after 48 h of incubation. *L. plantarum* strains isolated from sorghum decreased the pH of SBM from 6.47 to 3.93–4.03 (24 h) and 3.78–3.90 (48 h). *L. plantarum* strains obtained from maize reduced pH values of the MBM from 6.19 to 3.91–4.25 after 24 h, and then a negligible additional pH drop after 48 h of incubation was observed. pH values of control (non-inoculated FBM) remained unchanged along incubation for 48 h. Due to their capacity to low down pH, LpAv, LpA3, LpS13, and LpM15 were selected for further studies. These isolates were confirmed to belong to the species *L. plantarum* by sequencing of the 16S rRNA gene. This data are publicly available: https://www.ncbi.nlm.nih.gov/nuccore/MT799877 (LpA3), https://www.ncbi.nlm.nih.gov/nuccore/MT798595 (LpAv), https://www.ncbi.nlm.nih.gov/nuccore/MT798596 (LpS13), https://www.ncbi.nlm.nih.gov/nuccore/MT799876 (LpM15). RAPD profiling confirmed that these isolates were, indeed, different strains (Supplementary Figure 1).

**FIGURE 3:**
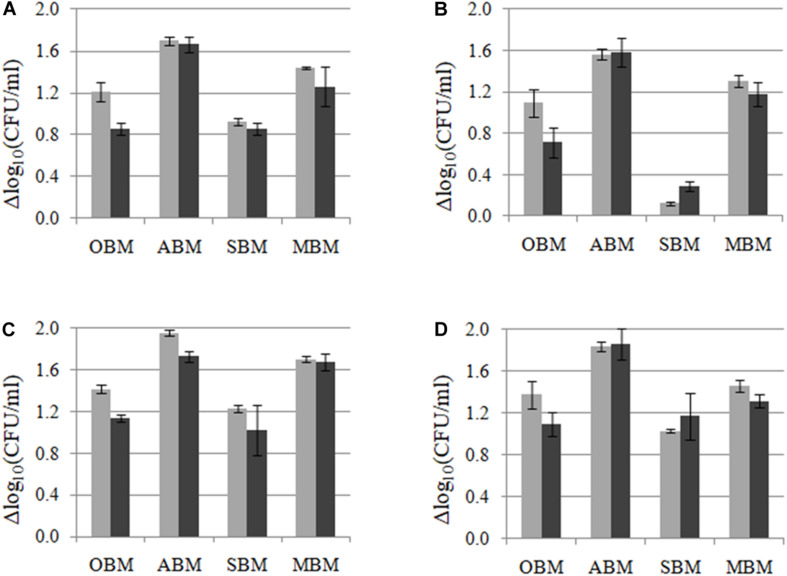
Growth (Δlog_10_ CFU/ml) of *L. plantarum* LpAv **(A)**, LpA3 **(B)**, LpS13 **(C)**, and LpM15 **(D)** in oat (OBM), lucerne (ABM), sorghum (SBM), and maize based medium (MBM), after 24 (■) and 48 h (■) of culture. Values are means (±SD) of three repetitions.

In order to study the capacity of *L. plantarum* strains to ferment different plant materials (others than the one from where they were isolated), crossed-growth kinetics and cell counts were performed for the four *L. plantarum* LpAv, LpA3, LpS13, and LpM15 in OBM, ABM, SBM, and MBM. Similar growth kinetics (figures not shown) were observed for the four strains on each plant material, but different cell counts were observed after 24 and 48 h of growth in each plant material (Figure 3). In terms of cell counts, the higher growth was supported by ABM (>1.6 Δlog_10_ CFU/ml), followed by MBM (1.2–1.4 Δlog_10_ CFU/ml), being *L. plantarum* A3 the strain that displayed the highest variability in growth capacity among the four FBM assessed. LpS13 displayed the highest growth capacity whereas the growth of LpAv was significantly lower in the four media (*p* < 0.05), compared to LpS13, particularly in SBM.

In case of pH (Table 2), the selected strains were more effective for fermenting maize and sorghum when compared to oat and lucerne. Although statistical differences were observed among pH values, these were very small and may be not relevant for practical purposes.

**TABLE 2:** pH values of forage-based medium (FBM) inoculated with *L. plantarum* strains (LpAv, LpA3, LpS13, and LpM15) at the beginning and after 24, 48, and 72 h of incubation.

FBM	Strain name	Time (h)			
		
		Before inoculation	24	48	72
Oat-based medium	Control	6.55 ± 0.01	6.56 ± 0.01^*a*^	6.65 ± 0.04^*a*^	6.64 ± 0.01^*a*^
	LpAv		4.68 ± 0.01^*b*^	4.92 ± 0.02^*b,c*^	4.96 ± 0.02^*b*^
	LpA3		4.71 ± 0.01^*c*^	4.91 ± 0.04^*b*^	4.87 ± 0.03^*c*^
	LpS13		4.77 ± 0.01^*d*^	5.01 ± 0.04^*b,c*^	4.99 ± 0.02^*b*^
	LpM15		4.79 ± 0.01^*d*^	5.02 ± 0.01^*c*^	4.99 ± 0.01^*b*^
Lucerne-based medium	Control	6.20 ± 0.01	6.18 ± 0.01^*a*^	6.27 ± 0.02^*a*^	6.16 ± 0.03^*a*^
	LpAv		5.77 ± 0.01^*b*^	5.91 ± 0.01^*b*^	5.88 ± 0.03^*b*^
	LpA3		5.68 ± 0.01^*c*^	5.74 ± 0.03^*c*^	5.74 ± 0.02^*c*^
	LpS13		5.68 ± 0.01^*c*^	5.86 ± 0.02^*b,c*^	5.82 ± 0.02^*b*^
	LpM15		5.78 ± 0.01^*b*^	5.94 ± 0.07^*b*^	5.96 ± 0.02^*d*^
Sorghum-based medium	Control	6.37 ± 0.03	6.44 ± 0.04^*a*^	6.43 ± 0.03^*a*^	6.38 ± 0.03^*a*^
	LpAv		3.97 ± 0.05^*b*^	3.98 ± 0.04^*b*^	3.85 ± 0.01^*b*^
	LpA3		4.03 ± 0.01^*b*^	3.96 ± 0.01^*b*^	3.87 ± 0.02^*b*^
	LpS13		3.95 ± 0.05^*b*^	3.94 ± 0.02^*b*^	3.86 ± 0.01^*b*^
	LpM15		3.93 ± 0.01^*b*^	3.95 ± 0.01^*b*^	3.89 ± 0.01^*b*^
Maize-based medium	Control	6.20 ± 0.03	6.24 ± 0.03^*a*^	6.20 ± 0.02^*a*^	6.19 ± 0.03^*a*^
	LpAv		3.93 ± 0.01^*b*^	3.81 ± 0.01^*b*^	3.74 ± 0.02^*b*^
	LpA3		4.03 ± 0.01^*c*^	3.83 ± 0.02^*b,c*^	3.74 ± 0.01^*b*^
	LpS13		4.07 ± 0.01^*c*^	3.88 ± 0.01^*c*^	3.77 ± 0.02^*c*^
	LpM15		4.04 ± 0.01^*c*^	3.83 ± 0.01^*b,c*^	3.78 ± 0.02^*c*^

*Values are means (±SD) of three repetitions. ^*a*,^^*b*,^^*c*^Values with different superscripts differ significantly (*p* < 0.05) in each column for the corresponding time and forage.*

#### Dose-Response of *L. plantarum* in Maize-Silage

**FIGURE 4:**
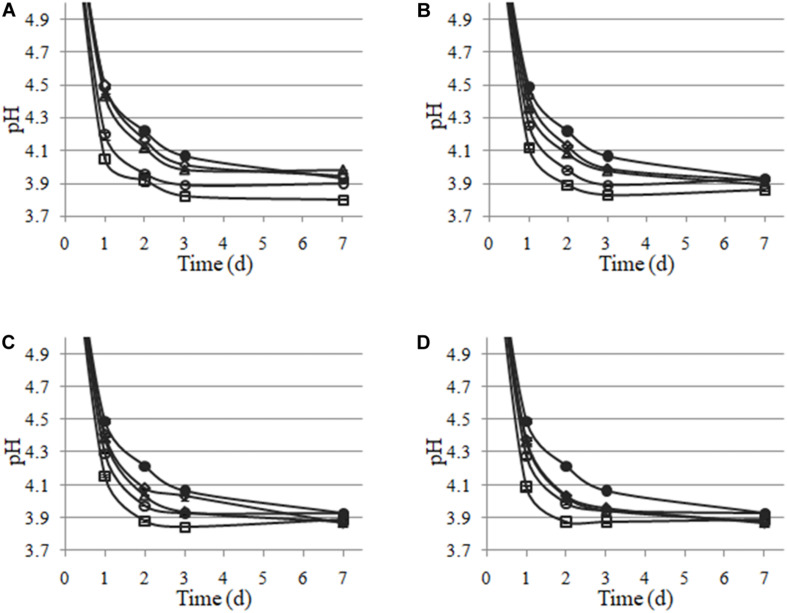
pH values of maize-silage uninoculated (🌑) and inoculated with *L. plantarum* LpAv **(A)**, LpA3 **(B)**, LpS13 **(C)**, and LpM15 **(D)**, to an initial concentration of 10^4^ (◆), 10^5^ (Δ), 10^6^ (○), or 10^7^ CFU/g (■). Values are means (± SD) of three repetitions.

**FIGURE 5:**
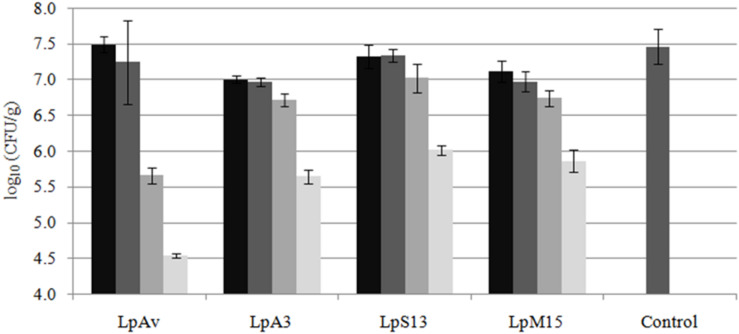
Total counts (log_10_ CFU/g) of LAB in maize-silage inoculated (or not: control) with 10^4^ (■),10^5^(■), 10^6^(■), and 10^7^(■) CFU/g of *L. plantarum* LpAv, LpA3, LpS13, or LpM15, after 30 days of fermentation at 25°C. Values are means (±SD) of three repetitions.

*Lactiplantibacillus plantarum* LpAv, LpA3, LpS13, and LpM15 were inoculated at four rates (10^4^, 10^5^, 10^6^, and 10^7^ CFU/g of fresh-chopped material) and fermentation was allowed to take place for 30 days. The pH values are shown in Figure 4. A statistical analysis (Dunnett test) was conducted after 1 day of fermentation. For LpAv, the dose of 10^4^ CFU/g produced no differences compared to control samples (*p* = 0.871), whereas pH achieved by the doses 10^5^, 10^6^, and 10^7^ CFU/g were significantly lower compared to control (*p* = 0.014, <0.001, and <0.001, respectively) and among them (*p* < 0.001, Tukey test). For LpA3, all doses induced a significant pH reduction (*p* < 0.05) and among them, doses were significantly different too (*p* < 0.01). For LpS13 and LpM15, all doses induced a significant reduction of pH when compared to control samples (*p* < 0.001); however, no differences were observed between the doses 10^4^ and 10^5^ CFU/g (*p* = 0.581 and 0.842, for LpS13 and LpM15, respectively). Comparing the performance of the different strains, no differences in pH were observed among them after 24 h of fermentation (*p* > 0.05). After 3 days of fermentation, the pH reached by LpS13 and LpM15 when inoculated at 10^5^ CFU/g was significantly lower to that observed in case of LpAv and LpA3 (*p* < 0.05). For the dose of 10^5^ CFU/g, differences were observed among all *L. plantarum* strains (*p* < 0.05). Finally, for the highest dose assessed, differences were observed between LpM15 and LpAv (*p* = 0.002) and LpA3 (*p* = 0.006). Furthermore, pH values of the silages after 30 days of fermentation were: 3.84 ± 0.04 for the control, 3.86 ± 0.17, 3.87 ± 0.12, 3.81 ± 0.15, and 3.81 ± 0.01, for *L. plantarum* LpAv, 3.88 ± 0.01, 3.88 ± 0.02, 3.88 ± 0.02, and 3.87 ± 0.02, for *L. plantarum* LpA3, 3.87 ± 0.03, 3.89 ± 0.02, 3.89 ± 0.04, and 3.90 ± 0.05, for *L. plantarum* LpS13 and 3.86 ± 0.02, 3.87 ± 0.05, 3.89 ± 0.04, and 3.89 ± 0.02, for *L. plantarum* LpM15, for the inoculation rates of 10^4^, 10^5^, 10^6^, and 10^7^ CFU/g, respectively. Differences were not significant in any case (*p* > 0.05). In terms of counts of total LAB in MRS agar, after 30 days of ensiling, it was observed that the higher the inoculation rate, the lower the total counts observed (Figure 5).

#### Crossed-Fermentation Capacity of *L. plantarum* Strains in Different Forages

*Lactiplantibacillus plantarum* LpAv, LpA3, LpS13, and LpM15 were able to grow in all laboratory-prepared forage-based liquid media (OBM, ABM, SBM, MBM), and all strains induced significant reductions in pH in a reasonable similar manner. In this context, their fermentative capacity was further studied in oat, lucerne, sorghum, and maize silages. Results of pH counts of total LAB and yeasts and molds are displayed in Table 3. In case of lucerne and sorghum silage, total counts on MRS on the day of inoculation (day 0), were significantly higher (ca. 1 log order CFU/g) than the total counts in control (non-inoculated) samples, suggesting that the difference was due to the counts of the *L. plantarum* strains inoculated. In oat and maize silages, epiphytic LAB at the beginning of the ensiling process (day 0), reflected by total counts on MRS agar, overcame the concentration of the inoculated *L. plantarum* strains used by more than 1 log cycle.

**TABLE 3:** Crossed-fermentation capacity of selected *L. plantarum* strains in oat, lucerne, sorghum, and maize silages.

Sample	Cell count (log_10_ CFU/g ± SD)						pH		
				
Lactic acid bacteria				Yeasts and filamentous fungi					
**Oat-silage**	***t* = 0 day**	***t* = 2 days**	***t* = 30 days**	***t* = 0 day**	***t* = 2 days**	***t* = 30 days**	***t* = 0 day**	***t* = 2 days**	***t* = 30 days**
Control	7.04 ± 0.04	9.20 ± 0.03^*a*^	8.07 ± 0.01^*a*^	7.57 ± 0.07	4.89 ± 0.46^*a*^	<2	6.33 ± 0.03	4.25 ± 0.03^*a*^	3.87 ± 0.02^*a*^
LpAv	7.15 ± 0.04	9.53 ± 0.10^*b,c*^	8.15 ± 0.13^*a*^	7.57 ± 0.07	4.62 ± 0.17^*a,b*^	5.43 ± 2.27	6.33 ± 0.03	4.08 ± 0.04^*b*^	3.89 ± 0.02^*a*^
LpA3	7.18 ± 0.05	9.44 ± 0.05^*b*^	7.99 ± 0.10^*a*^	7.57 ± 0.07	5.12 ± 0.39^*a*^	6.36 ± 2.24	6.33 ± 0.03	4.17 ± 0.08^*a,b*^	3.89 ± 0.02^*a*^
LpS13	7.19 ± 0.08	9.29 ± 0.01^*a*^	8.16 ± 0.06^*a*^	7.57 ± 0.07	3.70 ± 0.05^*b,c*^	<2	6.33 ± 0.03	4.05 ± 0.04^*b*^	3.92 ± 0.02^*a*^
LpM15	7.32 ± 0.08	9.58 ± 0.03^*c*^	8.71 ± 0.07^*b*^	7.57 ± 0.07	3.34 ± 0.51^*c*^	<2	6.33 ± 0.03	4.12 ± 0.03^*b*^	4.01 ± 0.04^*b*^
**Lucerne-silage**	***t* = 0 day**	***t* = 3 days**	***t* = 30 days**	***t* = 0 day**	***t* = 3 days**	***t* = 30 days**	***t* = 0 day**	***t* = 3 days**	***t* = 30 days**
Control	5.25 ± 0.18^*a*^	9.61 ± 0.12	8.58 ± 0.13^*a*^	5.07 ± 0.21	4.80 ± 0.19^*a*^	2.60 ± 0.30	6.28 ± 0.03	5.49 ± 0.24^*a*^	4.49 ± 0.03^*a*^
LpAv	6.64 ± 0.21^*b*^	9.64 ± 0.08	6.65 ± 0.17^*b*^	5.07 ± 0.21	4.20 ± 0.23^*b*^	3.06 ± 0.25	6.28 ± 0.03	4.84 ± 0.23^*b*^	4.39 ± 0.03^*b*^
LpA3	6.41 ± 0.18^*b*^	9.57 ± 0.07	6.55 ± 0.25^*b*^	5.07 ± 0.21	4.51 ± 0.23^*b*^	2.81 ± 0.16	6.28 ± 0.03	4.98 ± 0.19^*a,b*^	4.45 ± 0.04^*a,b*^
LpS13	6.47 ± 0.21^*b*^	9.75 ± 0.05	7.30 ± 0.25^*c*^	5.07 ± 0.21	4.04 ± 0.17^*b*^	2.88 ± 0.09	6.28 ± 0.03	4.91 ± 0.48^*a,b*^	4.42 ± 0.06^*a,b*^
LpM15	6.66 ± 0.18^*b*^	9.53 ± 0.23	7.03 ± 0.15^*b,c*^	5.07 ± 0.21	4.29 ± 0.11^*b*^	2.89 ± 0.11	6.28 ± 0.03	5.12 ± 0.39^*a,b*^	4.46 ± 0.03^*a,b*^
**Sorghum-silage**	***t* = 0 day**	***t* = 3 days**	***t* = 30 days**	***t* = 0 day**	***t* = 3 days**	***t* = 30 days**	***t* = 0 day**	***t* = 3 days**	***t* = 30 days**
Control	5.17 ± 0.10	8.78 ± 0.05^*a*^	7.49 ± 0.17^*a*^	5.33 ± 0.02	6.62 ± 0.24^*a*^	4.29 ± 0.02^*a*^	5.76 ± 0.04	3.89 ± 0.03^*a*^	4.19 ± 0.03^*a*^
LpAv	6.78 ± 0.08	9.45 ± 0.05^*b*^	<2.00	5.33 ± 0.02	7.03 ± 0.10^*b*^	4.50 ± 0.15^*a,b*^	5.76 ± 0.04	3.67 ± 0.01^*b,c*^	3.80 ± 0.01^*b,c*^
LpA3	6.72 ± 0.11	9.48 ± 0.01^*b*^	<2.00	5.33 ± 0.02	6.98 ± 0.03^*a,b*^	4.59 ± 0.14^*b*^	5.76 ± 0.04	3.69 ± 0.01^*b*^	3.84 ± 0.02^*b*^
LpS13	6.40 ± 0.01	9.48 ± 0.05^*b*^	<2.00	5.33 ± 0.02	6.81 ± 0.24^*a,b*^	4.70 ± 0.10^*b*^	5.76 ± 0.04	3.64 ± 0.01^*c*^	3.78 ± 0.02^*c*^
LpM15	6.64 ± 0.05	9.32 ± 0.02^*c*^	5.19 ± 0.16^*b*^	5.33 ± 0.02	6.68 ± 0.03^*a,b*^	4.66 ± 0.11^*b*^	5.76 ± 0.04	3.70 ± 0.01^*b*^	3.82 ± 0.02^*b,c*^
**Maize-silage**	***t* = 0 day**	***t* = 1 day**	***t* = 30 days**	***t* = 0 day**	***t* = 1 day**	***t* = 30 days**	***t* = 0 day**	***t* = 1 day**	***t* = 30 days**
Control	7.82 ± 0.08	9.16 ± 0.04	7.05 ± 0.12^*a*^	6.79 ± 0.10	Nd	3.86 ± 0.26^*a,c*^	5.96 ± 0.08	4.37 ± 0.02^*a*^	3.89 ± 0.02^*a*^
LpAv	8.21 ± 0.12	9.20 ± 0.05	6.52 ± 0.08^*b*^	6.79 ± 0.10	Nd	5.23 ± 0.17^*d*^	5.96 ± 0.08	4.17 ± 0.02^*b*^	3.89 ± 0.01^*a*^
LpA3	8.16 ± 0.09	9.09 ± 0.04	7.04 ± 0.03^*a*^	6.79 ± 0.10	Nd	4.35 ± 0.25^*b*^	5.96 ± 0.08	4.22 ± 0.01^*b*^	3.88 ± 0.01^*a*^
LpS13	8.27 ± 0.01	8.99 ± 0.07	6.68 ± 0.02^*b*^	6.79 ± 0.10	Nd	4.50 ± 0.04^*b*^	5.96 ± 0.08	4.19 ± 0.04^*b*^	3.83 ± 0.01^*b*^
LpM15	8.66 ± 0.18	9.09 ± 0.08	6.88 ± 0.04^*a*^	6.79 ± 0.10	Nd	3.76 ± 0.11^*c*^	5.96 ± 0.08	4.21 ± 0.03^*b*^	3.89 ± 0.01^*a*^

*Values are means (± SD) of three repetitions. ^*a, b, c*^Values in columns with different superscripts differ significantly (*p* < 0.05). Nd, not determined.*

#### Oat Silage

After 2 days of fermentation, LpAv and LpM15 silages showed a significantly higher (*p* = 0.001 and 0.038, respectively) count of total LAB (Δlog_10_ CFU/g = 2.37 ± 0.09 and 2.26 ± 0.03, respectively) compared to control (Δlog_10_ CFU/g = 2.15 ± 0.02). Moreover, significant lower pH was observed for LpAv, LpS13, and LpM15 silages compared to control samples. LpS13 and LpM15 were also effective for the early reduction of yeasts and molds.

Considering chemical analyses (Table 4), after 30 days of fermentation, ADF (%) and NDF (%) were significantly lower (*p* = 0.026 and 0.030, respectively) in LpM15 silage (33.37 ± 1.39 and 52.20 ± 2.02%) compared to control (35.53 ± 0.47 and 55.18 ± 0.40%).

**TABLE 4:** Chemical analyses of oat-silage inoculated with *L. plantarum* isolated from oat (LpAv), lucerne (LpA3), sorghum (LpS13), and maize (LpM15) at the beginning and after 30 days of fermentation.

Time (days)	Sample	DM (%)	DM loss (%)	CP (%)	NDF (%)	ADF (%)	LDA (%)	EE (%)	Ash (%)	N-NH3/NT (%)
*t* = 0	–	20.18 ± 0.30	–	11.75 ± 0.43	63.94 ± 0.37	35.81 ± 0.48	5.16 ± 0.24	3.41 ± 0.35	18.25 ± 0.43	Nd
*t* = 30	Control	19.72 ± 0.46	1.82 ± 0.39	11.80 ± 0.46	55.18 ± 0.40^*a*^	35.53 ± 0.47^*a*^	6.48 ± 0.15	3.77 ± 0.27	16.51 ± 0.27	0.93 ± 0.29
	LpAv	19.37 ± 0.24	2.05 ± 0.25	11.60 ± 0.42	53.80 ± 0.61^*a,b*^	34.63 ± 0.21^*a,b*^	6.07 ± 0.16	3.22 ± 0.06	15.56 ± 0.51	1.25 ± 0.29
	LpA3	19.65 ± 0.56	1.95 ± 0.28	12.09 ± 0.74	55.12 ± 1.78^*a,b*^	34.90 ± 1.40^*a,b*^	6.38 ± 0.08	3.44 ± 0.47	14.35 ± 2.82	1.13 ± 0.17
	LpS13	19.56 ± 0.56	1.92 ± 0.22	11.69 ± 0.46	53.59 ± 0.89^*a,b*^	34.10 ± 0.37^*a,b*^	5.94 ± 0.62	3.56 ± 0.28	15.47 ± 2.00	0.99 ± 0.20
	LpM15	19.85 ± 0.24	1.61 ± 0.50	12.75 ± 0.48	52.20 ± 2.02^*b*^	33.37 ± 1.39^*b*^	6.27 ± 1.01	3.61 ± 0.43	17.35 ± 1.96	0.94 ± 0.24

*Dry Matter (DM), DM loss, Crude Protein (CP), Neutral Detergent Fiber (NDF), Acid Detergent Fiber (ADF), Acid Detergent Lignin (LDA), Ether Extract (EE), Ammonia Nitrogen/Total Nitrogen (N-NH3/NT). Values are means (±SD) of three repetitions. ^*a, b, c*^Values in columns with different superscripts differ significantly (*p* < 0.05). Nd, not determined.*

#### Lucerne Silage

Although there were no significant differences in LAB counts between inoculated and control lucerne silage after 3 days of fermentation, the use of LpAv, LpA3, and LpS13 significantly decreased pH, compared to control samples (*p* < 0.05). In particular, LpAv also kept the pH significantly lower after 30 days (Table 3). All strains were able to significantly reduce the counts of yeasts and molds, compared to control. Furthermore, the ammonia-N content in LpAv (3.10 ± 0.73%), LpA3 (3.53 ± 0.47%), LpS13 (4.38 ± 0.09%), and LpM15 silages (4.68 ± 0.06%) were lower than for the untreated silages (6.53 ± 0.46%) after 30 days. LpM15 produced a significantly lower DM (32.32 ± 0.45%) than control (33.33 ± 0.45%) (Table 5).

**TABLE 5:** Chemical analyses of lucerne-silage inoculated with *L. plantarum* isolated from oat (LpAv), lucerne (LpA3), sorghum (LpS13), and maize (LpM15) at the beginning and after 30 days of fermentation.

Time (days)	Sample	DM (%)	DM loss (%)	CP (%)	NDF (%)	ADF (%)	LDA (%)	EE (%)	Ash (%)	N-NH3/NT (%)
*t* = 0	–	32.86 ± 0.31	–	21.83 ± 1.15	37.78 ± 1.35	26.53 ± 1.10	6.81 ± 0.30	2.10 ± 0.55	12.00 ± 0.98	Nd
*t* = 30	Control	33.33 ± 0.45^*a*^	0.53 ± 0.64	20.17 ± 1.40	36.31 ± 2.41	27.95 ± 3.44	8.28 ± 0.77	3.69 ± 0.82	13.26 ± 0.26^*a*^	6.53 ± 0.46^*a*^
	LpAv	33.19 ± 0.72^*a*^	−0.59 ± 0.60	20.68 ± 0.98	35.33 ± 0.65	26.50 ± 0.31	7.41 ± 0.10	2.63 ± 1.01	12.49 ± 0.42^*b*^	3.10 ± 0.73^*b*^
	LpA3	33.64 ± 0.34^*a,b*^	−0.30 ± 0.58	23.27 ± 3.02	36.38 ± 1.80	27.32 ± 2.22	7.87 ± 0.45	3.51 ± 0.37	12.82 ± 0.20^*a,b*^	3.53 ± 0.47^*b,c*^
	LpS13	33.01 ± 0.31^*a,b*^	−0.66 ± 0.62	20.38 ± 0.36	35.95 ± 1.21	27.28 ± 1.36	7.96 ± 0.06	4.43 ± 1.22	13.18 ± 0.14^*a*^	4.38 ± 0.09^*c*^
	LpM15	32.32 ± 0.45^*b*^	0.24 ± 0.68	20.02 ± 0.83	36.12 ± 1.55	30.87 ± 7.40	7.63 ± 0.24	3.52 ± 1.32	12.82 ± 0.14^*a,b*^	4.68 ± 0.06^*c*^

*Dry Matter (DM), DM loss, Crude Protein (CP), Neutral Detergent Fiber (NDF), Acid Detergent Fiber (ADF), Acid Detergent Lignin (LDA), Ether Extract (EE), Ammonia Nitrogen/Total Nitrogen (N-NH3/NT). Mean values ± SD are shown. Values are means (±SD) of three repetitions. ^*a, b, c*^Values in columns with different superscripts differ significantly (*p* < 0.05). Nd, not determined.*

#### Sorghum Silage

The total LAB counts (log_10_ CFU/g) in silages inoculated with all strains were significantly higher than in control samples. In addition, all four strains reduced the pH significantly faster than control. After 24 h the pH values were 4.20 ± 0.01 (LpAv, *p* < 0.001), 4.29 ± 0.05 (LpA3, *p* < 0.001), 4.26 ± 0.13 (LpS13, *p* < 0.001), 4.16 ± 0.02 (LpM15, *p* = 0.000) and 4.71 ± 0.04 (control). After 72 h of fermentation, pH of inoculated samples was still significantly lower than control samples (*p* < 0.001 in all cases, compared to control). Furthermore, after 30 days of fermentation, inoculated silages displayed a pH lower than 3.84, while the pH of the control was above 4. None of the strains were able to modify the chemical composition of silages after 30 days of fermentation, compared to control samples (Table 6).

**TABLE 6:** Chemical analyses of sorghum-silage inoculated with *L. plantarum* isolated from oat (LpAv), lucerne (LpA3), sorghum (LpS13), and maize (LpM15) at the beginning and after 2 and 30 days of fermentation.

Time (days)	Sample	DM (%)	DM loss (%)	CP (%)	NDF (%)	ADF (%)	LDA (%)	EE (%)	Ash (%)	N-NH3/NT (%)
*t* = 0	–	29.34 ± 0.22	–	6.61 ± 0.27	54.10 ± 0.76	30.38 ± 0.20	4.47 ± 0.90	1.34 ± 0.21	7.62 ± 0.14	Nd
*t* = 30	Control	26.79 ± 0.14	4.14 ± 0.95	6.39 ± 0.52	58.30 ± 1.38	32.91 ± 1.00	4.93 ± 0.38	3.63 ± 0.82	7.97 ± 0.31	1.24 ± 0.24
	LpAv	27.23 ± 0.33	4.56 ± 1.04	6.38 ± 0.21	56.26 ± 1.10	31.03 ± 1.48	4.66 ± 0.34	3.43 ± 0.59	7.84 ± 0.26	1.14 ± 0.17
	LpA3	26.87 ± 0.60	3.84 ± 0.83	6.72 ± 0.32	55.45 ± 1.33	30.14 ± 0.53	5.20 ± 1.16	3.60 ± 0.69	8.50 ± 0.57	1.12 ± 0.22
	LpS13	26.98 ± 0.21	3.85 ± 0.90	6.36 ± 0.19	56.54 ± 2.56	30.84 ± 2.15	4.80 ± 0.18	4.96 ± 0.57	7.88 ± 0.78	1.05 ± 0.08
	LpM15	27.51 ± 0.37	3.82 ± 0.28	6.73 ± 0.16	56.18 ± 4.44	31.62 ± 3.06	4.45 ± 0.17	4.13 ± 1.94	8.05 ± 0.89	1.10 ± 0.01

*Dry Matter (DM), DM loss, Crude Protein (CP), Neutral Detergent Fiber (NDF), Acid Detergent Fiber (ADF), Acid Detergent Lignin (LDA), Ether Extract (EE), Ammonia Nitrogen/Total Nitrogen (N-NH3/NT). Values are means (± SD) of three repetitions. Nd, not determined.*

#### Maize Silage

No differences were found in total LAB counts (CFU/g) among silos after 24 h of fermentation. However, the addition of *L. plantarum* strains significantly reduced pH after 24 h of ensiling. After 30 days of fermentation, pH values were lower than 3.90 in all cases, being the LpS13 silage the only one with a significantly lower pH value, compared to control. LpAv, LpS13, and LpM15 produced significantly lower ammonia-N content (4.56 ± 0.05, 4.51 ± 0.30, 4.91 ± 0.10%, respectively) compared to control (5.83 ± 0.11%), with no differences among inoculated silages (Table 7).

**TABLE 7:** Chemical analyses of maize-silage inoculated with *L. plantarum* isolated from oat (LpAv), lucerne (LpA3), sorghum (LpS13), and maize (LpM15) at the beginning and after 1 and 30 days of fermentation.

Time (days)	Sample	DM (%)	DM loss (%)	CP (%)	NDF (%)	ADF (%)	LDA (%)	EE (%)	Ash (%)	N-NH3/NT (%)
*t* = 0	–	37.42 ± 0.35	–	5.42 ± 0.04	35.91 ± 3.69	17.73 ± 1.91	1.64 ± 0.11	3.00 ± 0.16	4.40 ± 0.91	Nd
*t* = 30	Control	37.10 ± 0.86	1.12 ± 0.62	5.46 ± 0.03^*a*^	37.18 ± 2.98	21.41 ± 1.86	1.85 ± 0.16	3.94 ± 0.12^*a*^	4.59 ± 0.57^*a*^	5.83 ± 0.11^*a*^
	LpAv	37.49 ± 0.79	1.20 ± 0.25	6.09 ± 0.17^*a,b*^	34.32 ± 1.85	19.16 ± 1.09	1.73 ± 0.06	4.36 ± 0.12^*a,c*^	4.99 ± 0.16^*a,b,c*^	4.56 ± 0.05^*b*^
	LpA3	36.03 ± 0.94	1.66 ± 0.23	6.26 ± 0.35^*b,c*^	35.78 ± 4.31	19.16 ± 2.56	1.57 ± 0.45	5.45 ± 0.13^*b*^	5.41 ± 0.21^*b*^	5.71 ± 0.23^*a*^
	LpS13	37.44 ± 0.40	1.71 ± 0.70	6.12 ± 0.29^*a,b*^	34.28 ± 3.52	17.89 ± 1.94	1.48 ± 0.23	4.58 ± 0.21^*c*^	5.36 ± 0.05^*a,b,c*^	4.51 ± 0.30^*b*^
	LpM15	36.24 ± 0.25	1.56 ± 0.51	7.00 ± 0.43^*c*^	34.13 ± 1.98	18.03 ± 1.21	1.66 ± 0.12	4.02 ± 0.43^*a,c*^	4.49 ± 0.41^*a,c*^	4.91 ± 0.10^*b*^

*Dry Matter (DM), DM loss, Crude Protein (CP), Neutral Detergent Fiber (NDF), Acid Detergent Fiber (ADF), Acid Detergent Lignin (LDA), Ether Extract (EE), Non-Fibrous Carbohydrates (NFC), Ammonia Nitrogen/Total Nitrogen (N-NH3/NT). Values are means (± SD) of three repetitions. ^*a, b, c*^Values in columns with different superscripts differ significantly (*p* < 0.05). Nd, not determined.*

### Heterofermentative LAB

#### Selection of Heterofermentative LAB

So as to select the strains with the highest potential to control yeasts and molds, the production of acetic acid was screened among the heterofermentative isolates obtained (results not shown). The strains producing the highest amounts of acetic acid were selected for further studies: *L. brevis* LbB2 (6.39 ± 0.58 g/l), *L. brevis* LbM6 (6.89 ± 0.43 g/l), *L. fermentum* LfSY1 (5.87 ± 0.25 g/l), and *L. fermentum* LfM1 (7.56 ± 0.73 g/l). The concentrations of acetic acid produced by *L. buchneri* Ls141 and *L. buchneri* 463 were 5.42 ± 0.36 g/l and 5.30 ± 0.53 g/l, respectively.

#### Aerobic Stability in Maize-Silages

**FIGURE 6:**
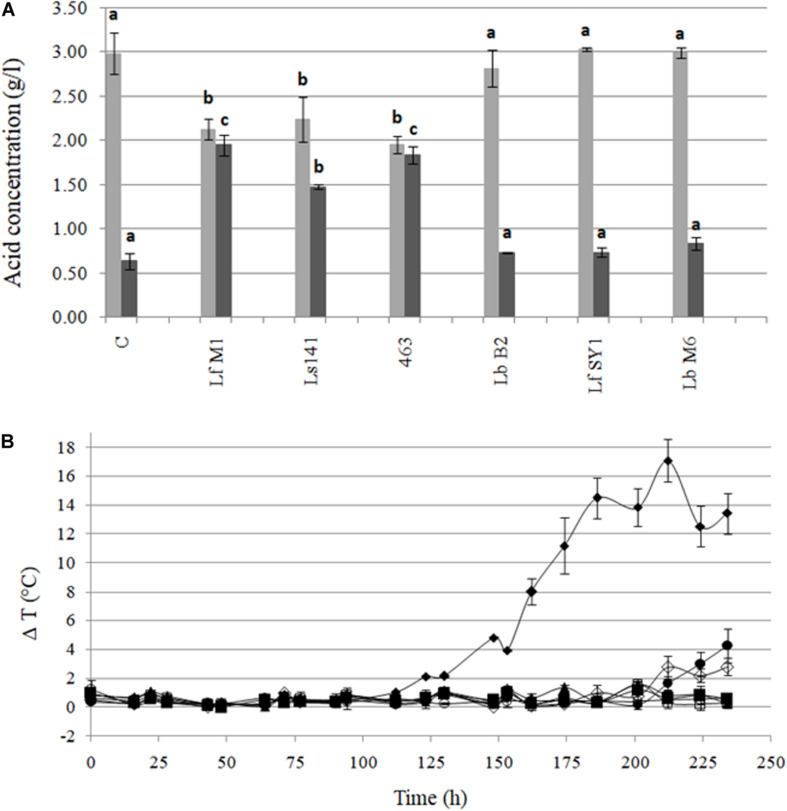
**(A)** Lactic (■) and acetic (■) acid content (g/l) of maize silage inoculated with heterofermentative strains after 90 days of fermentation. Values are means (±SD) of three repetitions. ^*a, b, c*^Values with different letter differ significantly (*p* < 0.05) in each determined organic acid. **(B)** Aerobic stability (ΔT°C = Tsilo-Tambient) of maize silages exposed to air after 90 days of fermentation. Untreated control (◆) and samples inoculated with *L. buchneri* 463 (▲), *L. buchneri* Ls141 (■), *L. fermentum* LfSY1 (🌑), *L. fermentum* LfM1 (○), *L. brevis* LbB2 (◆), and *L. brevis* LbM6 (□). Values are means (±SD) of three repetitions.

After 90 days of fermentation, pH values ranged from 3.85 to 4.08. The pH of silages inoculated with Ls141, 463, and LfM1 were significantly higher (*p* < 0.05) than the control silages. For these strains, significant higher concentrations of acetic acid were observed, being 2.3 – three times higher than in control samples (Figure 6A). When silos were manufactured, DM ranged from 42.6 ± 0.4% to 43.6 ± 1.5%, without significant differences (*p* > 0.05) among samples. After 90 days of fermentation, DM ranged from 38.9 ± 0.2% to 43.1 ± 3.4%, but again no significant differences were observed (*p* > 0.05) among treatments. Figure 6B shows the ΔT°C of the silos, exposed to the air after 90 days of fermentation, during 234 h (almost 12 days). Control samples lost aerobic stability (ΔT > 2°C) at the fastest rate (123 h, 5 days). In general, all strains under study were effective in conferring enhanced aerobic stability, with different capacity among them. The less effective strains were LbB2 (212 h, 8 days) and LfSY1 (224 h, 9 days), whereas the most effective ones were Ls141, 463, LfM1, and LbM6, which made silos stable at least for 12 days, when the experiment was stopped.

## Discussion

Epiphytic LAB widely vary in composition and numbers in plant materials (10^1^–10^7^ CFU/g), depending on many environmental factors (Pahlow et al., 2003). However, when favorable conditions of anaerobiosis, water activity, and temperature occur, spontaneous lactic acid fermentation may take place on the plant material, becoming LAB the dominants of the fermented substrate (Di Cagno et al., 2013). The strategy of inducing lactic fermentation to promote the growth of LAB has been largely used for the isolation of novel LAB candidates for their exploitation in forage fermentations, such as ensiling (Burns et al., 2018) and was the one used in this study. The homo- and hetero-fermentative species with the highest frequency of isolation were *L. pentosus/plantarum* and *L. brevis*, respectively.

*Lactiplantibacillus plantarum* is a species extensively used as inoculant for ensiling of lucerne (Filya et al., 2007; Zhao et al., 2020), sorghum (Thomas et al., 2013), maize (Dogi et al., 2013; Herrmann et al., 2015), ryegrass (Srigopalram et al., 2017; Muthusamy et al., 2020), and other forages (Ávila et al., 2010; Ni et al., 2015). In fact, *L. plantarum* is the most prevalent species in commercial inoculants (Fabiszewska et al., 2019). In our region, lucerne, oat, sorghum, and maize are the crops more frequently used for ensiling and then attention was focused on these crops. The growth ability of *L. plantarum* isolates was assessed in the same plant-based medium from which isolates were obtained (ABM, OBM, SBM, and MBM). Isolates obtained from the same plant material displayed different growth kinetics in laboratory-prepared media (FBM), indicating intrinsic diverse growth capabilities that justify strain screening. Considering that fast growth and acidification are of paramount importance for a microbial inoculants (Muck, 1988), *L. plantarum* LpAv, LpA3, LpS13, and LpM15 were selected for further studies after confirmation of their identity (partial 16S rRNA sequencing) and diversity (RAPD).

A common claim in commercial inoculants is that strains isolated from the same plant material where they are going to be used may perform better than strains isolated from other substrates (Weinberg, 1996). Crossed-growth kinetics and cell counts were performed for the four strains in OBM, ABM, SBM, and MBM. Considering the results obtained in a comprehensive way (growth kinetics, cell counts, and pH), it was observed that growth capacity was related to the nature of the plant material rather than to the origin of the strain itself, confirming that for an homofermentative species as *L. plantarum*, inoculant’s success depends on adequate substrate availability (Muck, 1988).

The fermentative capacity of the four *L. plantarum* strains were then assessed in maize, the main forage material use for ensiling in Argentina and worldwide (Weinberg and Ashbell, 2003; Khan et al., 2015), at four inoculation rates (10^4^, 10^5^, 10^6^, and 10^7^ CFU/g). Fast acidification is a valuable characteristic for inoculants to preserve the silage by both inactivating plant proteases and reducing the growth of pathogenic and spoilage microorganisms (Dunière et al., 2013; Queiroz et al., 2018). Although statistical differences were observed for pH when strains were compared among them, these differences were negligible in terms of practical applications. The biggest magnitude of differences in pH were observed for the different doses rather than for the different strains. Therefore, and considering acidification capacity, it can be reasonable to assume that all four *L. plantarum* strains assessed were able to ferment maize in a reasonably similar manner. It is interesting to note that the lowest the inoculation rate, the higher the total counts of LAB observed after 30 days of fermentation. Our hypothesis, yet to be proved, is that extensive metabolic adaptation may have occurred when the lowest inoculation rate was used (10^4^ CFU/g). This low inoculation rate certainly implied more duplication cycles until reaching the stationary phase, than an inoculation rate of 10^7^ CFU/g. The latter concentration is closer to the one commonly reached by LAB at the stationary phase (ca. 10^8^ CFU/g), then less duplication cycles are needed to reach the stationary phase, and in consequence less time is available to develop resistance due to exposure to environmental stresses. This adaptation is crucial for survival because it stimulates the production of additional energy and lowers the stress level, e.g., through alkalization of the cytosol under acidic conditions (Papadimitriou et al., 2016). These results also allow to confirm that the strains, even at the lowest inoculation rates, were able to dominate the fermentation by inhibiting the proliferation of epiphytic microbiota. For example, in samples inoculated with 10^7^ CFU/g, total counts after 30 days of fermentation were fairly low, suggesting that the strains quickly reached the stationary phase, impairing the proliferation of the epiphytic microbiota, and entered the dead phase. In other words, the inoculated strains did not allow the growth of the epiphytic microbiota, as can be observed for control samples.

Considering that significant differences compared to control were observed for all strains for a dose of 10^5^ CFU/g, and that inoculating at doses as high as 10^7^ CFU/g is economically not feasible (Oliveira et al., 2017), the dose of 10^6^ CFU/g was chosen for further studies. This dose was selected to ensure a proper fermentation since the epiphytic microbiota carried by different forage crops, or even by different harvests of the same plant material, may vary depending on several factors such as the type of plant, maturity of the crop, epiphytic bacteria content, and environmental factors like weather and harvesting conditions (Lindow and Brandl, 2003; Di Cagno et al., 2013).

*Lactiplantibacillus plantarum* LpAv, LpA3, LpS13, and LpM15, which were able to grow in all four FBM, were used to determine their fermentative capacity in oat, lucerne, sorghum, and maize silages.

LpS13, derived from sorghum, and LpM15, derived from maize, were effective strains for the acidification of oat silage and were both also able to control yeasts and molds. In addition, considering the reduction in ADF and NDF achieved by LpM15, as both chemical components are indirectly related to digestibility and DM feed intake, respectively, the reduction observed for these parameters could promote beneficial effects on animal performance (Meeske et al., 2002; Fulgueira et al., 2007). The level of epiphytic total LAB in the fresh plant material was close to that of the inoculated *L. plantarum* strains. Therefore, inoculated LAB could be potentially overwhelmed by the epiphytic population, being this fact an additional challenge for the selected strains to dominate the fermentation (Filya et al., 2007). In spite of this challenge, the fermentation process was accelerated by the inoculation of LpAv, LpS13, and LpM15, and silage quality was improved by the latter.

The use of LpAv in lucerne silage significantly decreased pH, and also kept the pH significantly lower after 30 days. Furthermore, the ammonia-N content was lower in all cases, compared to untreated silages, which can suggest less proteolysis and deamination rate. Particularly, the ammonia-N concentration was two times lower in LpAv silages compared to control. This parameter is generally used as an indicator of silage protein degradation (Whiter and Kung, 2001). The lower DM induced by LpM15 use, which might be due to a slow pH drop, is an unfortunate event as it could lead to a DM lost (Thomas et al., 2013). Ensiling lucerne may be challenging because of the low content of water-soluble carbohydrates and its high buffering capacity (Pang et al., 2011). However, *L. plantarum* has been successfully used over a wide range of DM, pH values reported after fermentation ranged from 4.29 to 5.01 (Filya et al., 2007; Ogunade et al., 2016). In general, the inoculation with homofermentative strains may render beneficial effects on the fermentation process (Whiter and Kung, 2001). In previous reports, a mean DM after ensiling, slightly lower than the initial mean value, was observed (Whiter and Kung, 2001; Rizk et al., 2005; Liu et al., 2016). This may occur when initial and final individual values are close among them. Then, the differences of the means may be negative, but not statistically significant, and so the loss of DM may look negative. When a difference is not statistically significant, then we cannot conclude that a negative loss of DM was indeed an increase of DM during ensiling, there was just preservation of DM.

In case of sorghum silages, after 30 days of fermentation, inoculated samples displayed a pH lower than the control group. Although inoculation did not modify the chemical composition of silages after 30 days of fermentation, compared to control samples, all *L. plantarum* strains improved the ensiling process, not only by accelerating the pH drop by day 3, but by leaving a final pH significantly lower than control. Despite all strains were suitable for sorghum ensiling, significant amounts of viable LAB were found after 30 days of fermentation only when LpM15 was used. In a recent work (Alhaag et al., 2019), two *L. plantarum* strains were employed for sorghum silages (a commercial one and another isolated from sorghum) and, although both strains preserved the silages overall quality, the commercial strain was not found viable after 30 days of ensiling. Viable LAB in silages may display a potential probiotic role for ruminants (Acosta Aragon, 2012; Ellis et al., 2016; Fabiszewska et al., 2019), an hypothesis that needs further studies, then viability along storage may be considered a potential advantage.

It is generally considered that a fast decrease of pH close to 4.2 or less is adequate for maize preservation. These pH values are commonly achieved in the first hours of fermentation, even without the use of microbial inoculants, so there may not be too much room for accelerating the process (Nkosi et al., 2011). In this work, no differences were found in total LAB counts (CFU/g) among silos after 24 h of fermentation. However, the addition of *L. plantarum* strains significantly increased the rate of pH drop compared to control samples. All *L. plantarum* strains may be regarded as equally effective for decreasing the pH of maize silage. These results agreed with the previous dose-response assay in maize, in line with the *in vitro* to *in situ* correlation observed by Saarisalo et al. (2007). LpAv, LpS13, and LpM15 produced significantly lower ammonia-N content, compared to control samples. Similar to oat silage, these improvements in the nutritional value and pH decrease of the forage can be observed despite the fact that epiphytic LAB content in fresh maize was higher than the inoculation rate.

The performance of four *L. plantarum* strains isolated from oat, lucerne, sorghum, and maize was compared in these four plant materials, making all possible combinations, what we called crossed-fermentation. LpM15 derived from maize was the most effective strain for oat ensiling and would be the strain of choice for sorghum silage, whereas LpAv, isolated from oat, showed a better performance than the other strains in lucerne, both accelerating the pH drop and decreasing the proteolysis process. All *L. plantarum* strains may be regarded as equally effective for decreasing the pH of maize silage, but in particular LpAv, LpS13, and LpM15 produced significantly lower ammonia-N content, compared to control samples. Our results support the fact that the origin of the strain is of secondary importance, being its own ensiling capacity and the silage material characteristics what matters (Ávila et al., 2011).

*Lentilactobacillus buchneri* is presently the gold standard to promote aerobic stability in corn silage (Santos et al., 2013; Da Silva et al., 2014) or Napier grass (Guan et al., 2020). Acetic acid is one of the main organic acids produced by heterofermentative LAB and it has the capacity of promoting aerobic stability when silos are opened (Danner et al., 2003). *L. brevis* LbB2, *L. brevis* LbM6, *L. fermentum* LfSY1, and *L. fermentum* LfM1 were found to be the highest acetic acid producers.

Once silos are opened, fermented material is exposed to air and yeasts fermentation may occur. Aerobic stability is the capacity of silage in maintaining the sanitary and nutritive value of the ensiled forage for a longer period of time, in the presence of air. In our study aerobic stability was considered lost when the temperature of the ensiled material was 2°C above the room temperature (Burns et al., 2018). After 90 days of fermentation, the silages were well preserved, based on their pH and DM values. Control silages lost aerobic stability after 5 days of being exposed to air, followed by those inoculated with *L. brevis* LbB2 (8.8 days) and *L. buchneri* 463 (9.3 days), whereas the rest of the strains were effective in maintaining the inner temperature until the end of the assay (9.75 days). Although *L. buchneri* is the main species used in commercial inoculants to improve aerobic stability, some strains may produce biogenic amines, which is an undesirable characteristic (Nishino et al., 2007) that justify the research of other heterofermentative LAB (Muck et al., 2018). There are some reports which show that *L. brevis* enhanced the acetic acid content and aerobic stability of maize silages compared to control but less efficiently than *L. buchneri* (Danner et al., 2003; Acosta Aragón et al., 2012). Wild strains of *Lactobacillus hilgardii* were proposed for this aim too (Reis et al., 2017). On the other hand, there is limited evidence published about the possibility of using *L. fermentum* for enhanced aerobic stability (Adesogan et al., 2003). To the best of our knowledge, *L. fermentum* has never been employed in commercial inoculants as aerobic stability promoter in the southern cone. In this sense, the results of this study show a promising future for *L. fermentum* LfM1, among other isolates, to be used as silage inoculant for enhanced aerobic stability. This fact can contribute to the development of domestic inoculants to contribute to import substitution industrialization.

## Conclusion

In this study the ubiquitous presence of LAB on 14 out of 15 plant materials analyzed was shown. From more than 100 isolates obtained, the application of a succession of experiments allowed us to narrow down the number of potential candidates of silage inoculants to two strains. Based on the studies performed, *L. plantarum* LpM15 and *L. fermentum* LfM1 displayed potential to be used as inoculants, however further studies are needed to determine their performance when inoculated together. The former because it positively influenced different quality parameters in oat, lucerne, sorghum, and maize silage, and the latter because of its capacity to confer enhanced aerobic stability to maize silage. The rest of the strains constitute a valuable collection of autochthonous strains that will be further studied in the future for new applications in animal or human foods.

## Data Availability Statement

The datasets presented in this study can be found in online repositories. The names of the repository/repositories and accession number(s) can be found in the article/Supplementary Material.

## Author Contributions

MP and GV designed the study, analyzed the data, and contributed to the writing of the manuscript. MP led all the experiments. JO was responsible for the HPLC analysis. AB and MP performed the molecular identification of the strains. MG and AM performed the chemical analyses. GV and AB participated in the revision of the manuscript and scientific discussions. All authors contributed to the article and approved the submitted version.

## Conflict of Interest

The authors declare that the research was conducted in the absence of any commercial or financial relationships that could be construed as a potential conflict of interest.

## Footnotes

1 https://blast.ncbi.nlm.nih.gov/Blast.cgi

## Supplementary Material

Supplementary Figure 1 RAPD profiles with primers B10 and M13 for *L. plantarum* strains isolated from lucerne, oat, sorghum and maize. Positive control: *L. plantarum* Ls71.
