# Supplementary figures and images for: Potential of Lactic Acid Bacteria Isolated From Different Forages as Silage Inoculants for Improving Fermentation Quality and Aerobic Stability
Source: Front Microbiol. 2020 Dec 8;11:586716. doi: 10.3389/fmicb.2020.586716 (PMC7752947; doi:10.3389/fmicb.2020.586716)

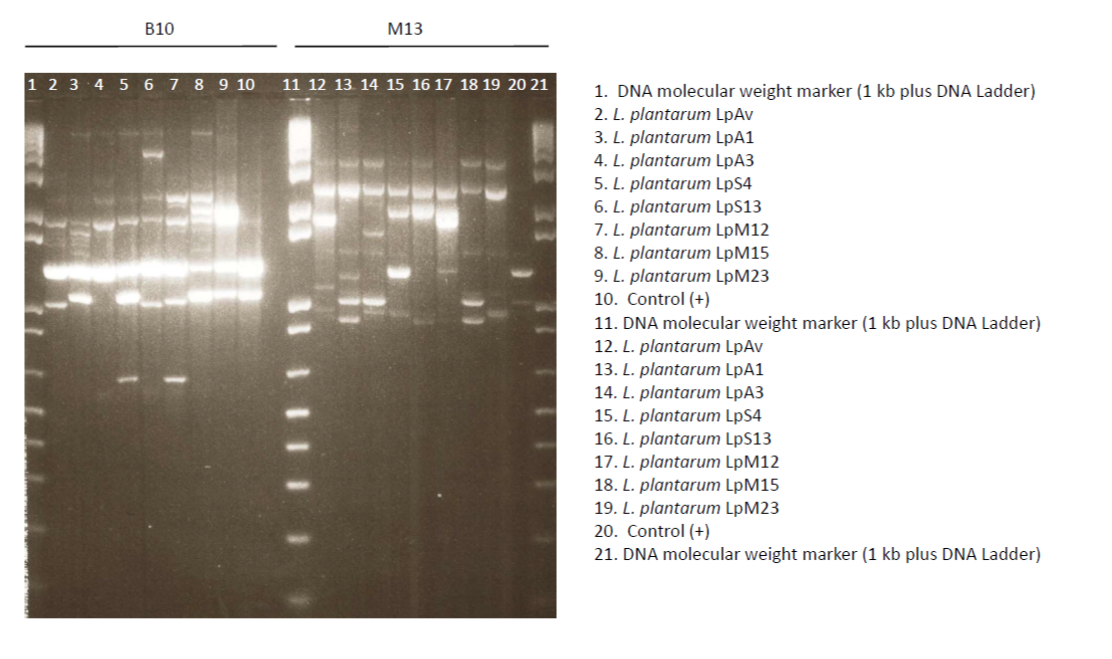

Supplement: Supplementary Figure 1 — RAPD profiles with primers B10 and M13 for L. plantarum strains isolated from lucerne, oat, sorghum and maize. Positive control: L. plantarum Ls71. [file Image_1.TIF]
